# Perception of District Judges and Lawyers Towards Medico-legal Reports, Medical Certificates and Medical Expert Opinion

**DOI:** 10.31729/jnma.3410

**Published:** 2018-08-31

**Authors:** Nuwadatta Subedi, Hima Raj Giri

**Affiliations:** 1 Department of Forensic Medicine, Gandaki Medical College Teaching Hospital, Pokhara, Kaski, Nepal; 2 Department of Law, Prithvi Narayan Campus, Pokhara, Kaski, Nepal

**Keywords:** *autopsy*, *expert testimony*, *forensic expert*, *medical witness*, *rape*

## Abstract

**Introduction:**

The medico legal reports and certificates prepared by doctors can be used as valuable documentary evidence in the court of law. The study was designed with objectives to explore the perception of judges and lawyers about the quality of medico legal reports prepared by the doctors and their competence in providing the expert evidence in the court.

**Methods:**

It is a questionnaire based cross sectional study conducted among the district judges and government attorneys of 75 districts of Nepal from March to May 2016. The data obtained was analysed by SPSS version 16.0.

**Results:**

Among a total of 78 participants who responded the questionnaire, 40 (51.3%) were district judges and 38 (48.7%) district attorneys. Most of them graded that the reports prepared by the doctors were just average. Among them, 49 (63.6%) strongly agreed and 28 (36.4%) partially agreed that the reports were useful in deciding the cases. A total of 44 (56.4%) respondents strongly agreed and 34 (43.6%) partially agreed that expert opinion of the doctors in the courts were useful to decide the cases. Seventy one (92.2%) of them rated general doctors as moderately competent.

**Conclusions:**

The medical reports prepared by the Nepalese doctors were just average as perceived by judges and lawyers and the competency in presenting the evidence in courts was moderate as rated by them.

## INTRODUCTION

Medical doctors should also be competent in medico legal matters as it is one of their responsibilities to the state.^1^ The reports and certificates are used as valuable documentary evidence in the courts. Therefore, they have to be clear, unambiguous, understandable and help the legal authorities in deciding the cases.^2^

The challenges to the expert witness in legal systems can be found in literature.^3–5^ But that provided by Professor Roy Meadow in the UK is an important and exemplarity case.^6^ The issue of competence of Nepalese doctors in recording medical evidence and presenting it in the courts has not been analysed so far.

The study was conducted to explore the perception of judges and lawyers about the quality of medico-legal reports and medical certificates prepared by the doctors and their competence in providing oral testimony of the evidence in the courts.

## METHODS

**Figure 1.**
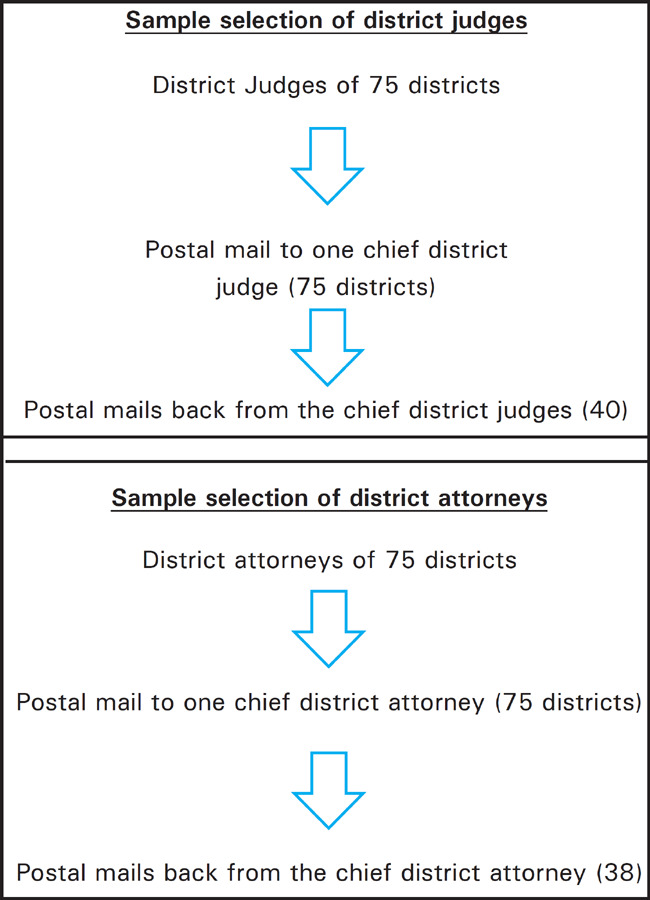
Flow chart of selection of Participant.

It is a cross-sectional, descriptive study conducted in the Department of Forensic Medicine, College of Medical Sciences, Bharatpur, Nepal from March to May 2016. The study was ethically approved from Institutional Review Committee of College of Medical Sciences. Those respondents who voluntarily consented to participate in the study were included. Convenience sampling technique was used where the chief judges and government lawyers of all the then 75 districts of Nepal were included (Figure 1). All the available respondents were taken. The questionnaire was mailed by post to the district judges in the courts and the government attorneys of all the 75 districts. The envelope contained a letter addressing the proper authorities regarding the purpose of the study, the researcher's introduction and the way of answering the questionnaire were mentioned. The postal ticket and envelope with address of the investigator were also included so that the respondents didn't have to pay to mail the filled questionnaire back.

A standard structured questionnaire was designed to collect the data. Some of the components of the questionnaire were prepared on the basis of previously published study,^3^ discussion among the reputed faculty of Law, lawyers, judges and doctors. The questionnaire consisted of items to evaluate the quality of the medico-legal reports prepared by the doctors and their competency in the courts of the law. Attempt was made regarding the comparison of the competence of the forensic medical experts and the general medical practitioners. The last question was an open question which asked any general comments to the medical practitioners in legal matters so that if the respondents felt to add something in the issue, could include them.

The quality of the reports was evaluated as “excellent”, “average” and “poor” while the competence of the doctors was graded as “highly competent”, “moderately competent” and “incompetent”. The usefulness of the reports and the oral testimony of experts was evaluated as “strongly agree”, “moderately agree” and “disagree”. The questionnaire was pretested among 20 private lawyers of Chitwan district and the discrepancies corrected before using them to the study population.

The data obtained was entered in Mircosoft Excel and analysed by SPSS version 16.0. The reliability of the multiple Likert questions was tested by Cronbach's alpha. In our analysis, Cronbach's alpha was 0.723 and thus considered reliable.

## RESULTS

Only one of the respondents was female. The median age of the respondents was 47 years (Interquartile range: 10.50). The mean experience in legal field was 21.52 years (SD: 7.59, Range: two to 36 years). The participants of the study included 38 (48.7%) district attorneys and 40 (51.3%) district judges. All of them responded they had had used medical certificates/ medico-legal reports in their legal practice in their career. The use of various kinds of medico-legal reports and certificates by the lawyers and judges is shown (Table 1).

**Table 1:** Use of medico legal reports (MLRs) and medical certificates by the legal authorities.

Have you encountered the following medical certificates/medico-legal reports in your legal practice?	District Judges		Govt Lawyers	
	Yes n (%)	No n (%)	Yes n (%)	No n (%)
Report on victim of rape (respondents= 78)	40 (100.0)	0 (0.0)	38 (100.0)	0 (0.0)
Report on accused of rape (respondents= 78)	39 (97.5)	1 (2.5)	37 (97.4)	1 (2.6)
Injury report (respondents = 78)	40 (100.0)	0 (0.0)	38 (100.0)	0 (0.0)
Post mortem report (respondents = 78)	40 (100.0)	0 (0.0)	38 (100.0)	0 (0.0)
Age report (respondents = 78)	37 (92.5)	3 (7.5)	38 (100.0)	0 (0.0)
Drunkenness report (respondents = 77)	30 (76.9)	9 (23.1)	36 (94.7)	2 (5.3)
Fitness certificate (respondents = 74)	19 (50.0)	19 (50.0)	20 (55.6)	16 (44.4)
Psychiatric assessment (respondents = 73)	17 (46.0)	20 (54.0)	25 (69.4)	11 (30.6)
The percentage in the subgroups has been calculated for district judges and lawyers separately.				

Table 2 shows 49 (63.6%) of the respondents strongly agreed that the medico legal reports were useful to decide the cases.

**Table 2:** Usefulness of medico-legal reports in deciding cases (n = 77).

Usefulness of MLRs	District Judges n	Govt Lawyers n	Total n (%)
Strongly agree	22	27	49 (63.6)
Moderately agree	17	11	28 (36.4)
Disagree	0	0	0 (0.0)

The quality of reports of victim of rape was average as judged by the 67 (85.9%) respondents. Only eight (10.2%) graded them as excellent and three (3.8%) as poor. When the quality of the reports of accused of rape was asked, the responses were average in 68 (87.2%), as judged by the respondents. Only five (6.4 %) graded them as excellent and poor each. The respondents were asked to reveal the quality of injury reports drafted by the doctors. Majority 60 (76.9%) responded that the reports were average. The injury reports were rated as excellent by 13 (16.7%) and poor by five (6.4%). The quality of autopsy reports was judged by 17 (21.8%) respondents as excellent, 54 (69.2%) rated them as average, seven (8.9%) replied that they were poor. The quality of age reports prepared by the doctors were excellent as reported by the 12 (15.8%) respondents, 57 (75%) reported them as average and seven (9.2%) as poor. All the lawyers and judges had encountered general doctors and 42 (53.8%) of them forensic experts in the courts for oral testimony regarding the reports they had prepared. Table 3 shows the usefulness of oral testimony of the evidence by doctors in the court

**Table 3:** Usefulness of oral testimony of the evidence by doctors in the court. (N = 78)

Usefulness	District Judges n	Govt Lawyers n	Total n (%)
Strongly agree	25	19	44 (56.4)
Partially agree	15	19	34 (43.6)
Disagree	0	0	0 (0.00)

**Table 4:** Competence of general medical witness in the court of law (n=77).

Competence	District Judges n	Govt Lawyers n	Total n (%)
Highly competent	3	1	4 (5.2)
Moderately competent	35	36	71 (92.2)
Incompetent	1	1	2 (2.6)

The competence of general medical (medical officers and doctors of other specialties other than forensic medicine) and of forensic medical witness in the courts of law is presented in table 4 and 5 respectively.

**Table 5:** Competence of forensic medical witness in the court of law (n=44).

Competence	District Judges n	Govt Lawyers n	Total n (%)
Highly competent	11	13	24 (54.6)
Moderately competent	10	10	20 (45.4)
Incompetent	0	0	0 (0)

Fifteen (19.2%) of the participants replied that they always had problems in interpretation and understanding of the reports written by the doctors. Sixty-three (80.8%) reported that they sometimes had the problem. There were no any respondents who replied that they never have problems in interpreting the reports.

## DISCUSSION

The present study has explored the view of the judges and lawyers regarding the expert medical evidence in the form of medico legal reports and certificates and the testimony in the courts of law. Only one respondent was a female lawyer. This indicates less participation of females in the legal field in government sector. Women are more feminist conscious and that this can be implied that females' involvement in legal field can be better for the legal institution.^7^ An analysis has revealed that the female participation is also less in the USA.^8^

All respondents had used medical expert evidence during their legal practice. The use of medical expertise in the courts is now inevitable. As mentioned by De Renzi,^9^ “the relations among medical practitioners and with legal authorities, provide a hitherto neglected context within which to understand contemporary epistemological debates, from claims and challenges to expertise to the definition and production of evidence, including the status of signs, personal observation and tests.” This also implies to our study that most of the respondents have strongly agreed that the medico legal reports were important to decide the cases. None of them deferred that they are not useful. But as the medical officers routinely involved in preparing medico legal reports are not much trained, the reports could not be that satisfying to the legal authorities. As opined by Knight et al,^10^ “A poor opinion is often worse than no opinion at all, as in the latter case, the legal authorities will at least be aware of the deficiency in their evidence, rather than be misled by the often dogmatic inaccuracies of an inexperienced doctor.” Therefore, any medical officer, who is employed on this duty, should be adequately trained before handling this job. The respondents had mentioned that sometimes medical officers write rape is done or attempt to sex. The respondents even have suggested that doctors should write the findings and the court will decide. Statement like: there is evidence of recent sexual activity, should be written. Inconclusive reports are given in sexual assault examination. Thus, there is improvement in the part of the medical officers in preparing such reports and it has also been pointed out by the legal personnel. Many of the respondents have commented that the injury reports are inconclusive and fatality is not clearly mentioned. Sometimes the reports are ambiguous on that the injuries are opined simple but fatal. Some have even alleged that reports don't seem to be reliable, seem to be influenced by police reports. The medical officers need to improve on preparing the injury reports so that proper decision could be arrived in the courts of law. They should interact with the legal authorities what constitutes fatalities in relation to the injuries. The legal authorities should also understand that the human body is very unpredictable, and the situation can also turn abruptly in some injured individuals. The injury is called fatal if it is “sufficient in the ordinary course of nature to cause death.”^11^ Hence categorizing those injuries should be done meticulously and it can be improved with experience.

Post mortem reports are one of the frequently prepared Medico legal reports by the doctors and are very important as they are vital in death investigation process. There are so many shortcomings in the mortuaries of Nepal and autopsies are just restricted mostly to formalities. The medical officers inadequately trained can't perform quality examination and provide quality reports. Hence, the forensic experts should be employed by the state to improve the standards of the autopsies. Many claimed that the age should not be given in range and exact age should be given, which is almost impossible with the present medical expertise and aids.^11^ Still, the doctors could improve in providing the age in as narrow range as possible to help the legal authorities. As per section 23 (7) of the Evidence act of Nepal, 1974, “any opinion expressed by a person may be taken as evidence only if he/she appears before the court in person as a witness.” Thus, the doctors can be summoned in the courts of law to testify their opinions. But, in accordance to section 18 (a) of evidence act, “In case, there is no issue between the parties to the case on the facts mentioned by the expert in the Post-mortem Report or the fact so mentioned is not inconsistent with the evidence if any, such fact may be taken, as evidence even if the expert does not appear before the court.”^12^

But whenever the court requires, the doctors should attend the court to testify and elaborate their reports. When the doctors have prepared any medico legal reports, they should be ready to attend the courts and they should understand that their opinion can be very valuable and taken as evidence to decide the cases. Most of the respondents have rated the competency of the general doctors in the courts as just average. As the medical officers have to perform their general medical duties, they can't focus more on their legal duties. Hence the rating could be so. While the forensic medical witnesses were judged as highly competent by more of the respondents. The forensic practitioners are destined to serve the legal authorities; the rating seems obvious. To improve the quality of medical expert witness, more medico legal works should be designated to the forensic experts. A similar study from Sri Lanka has also highlighted on the expectations of judiciary from the expert medical witness.^3^ The courts and the lawyers have problems in understanding the technical language in the reports provided by the doctors and hence pose a serious problem in proper administration of justice. The doctors should prepare the reports in clear legible language. Most of the respondents have highlighted on this section in their open comments. Doctors should write the reports in simple non-technical language to minimize ambiguity. It can also avoid many calls of doctors in the courts to testify and interpret their reports.

The medico legal facilities provided to the hospitals should be strengthened. It is one of the most neglected part of the hospitals throughout the country. Forensic experts are the doctors destined to perform medico legal works. Unfortunately, there are not much forensic experts in Nepal and the experts are mainly working in medical colleges and restricted in teaching the medical students. The experts should be engaged by the government to perform medico legal duties. The major hospitals should establish a forensic department headed by forensic practitioner.

As convenience sampling method was used in this study, there could have been some selection bias of the research participants. This is the limitation of our study and can be overcome by using probability sampling in future studies.

## CONCLUSIONS

Majority of the respondents strongly agreed that the medico legal reports were useful in deciding the cases in the court and those drafted by the doctors were just average or even poor.

Majority of respondents also reported that the expert opinion provided by the doctors in the courts were highly useful to decide the cases. The competency of the forensic experts was rated high and of the general medical practitioners was just average.
